# Automatic Round-the-Clock Detection of Whales for Mitigation from Underwater Noise Impacts

**DOI:** 10.1371/journal.pone.0071217

**Published:** 2013-08-12

**Authors:** Daniel P. Zitterbart, Lars Kindermann, Elke Burkhardt, Olaf Boebel

**Affiliations:** 1 Ocean Acoustics Lab, Alfred-Wegener-Institut Helmholtz-Zentrum für Polar- und Meeresforschung, Bremerhaven, Germany; 2 Department of Physics, University of Erlangen-Nuremberg, Erlangen, Germany; Aristotle University of Thessaloniki, Greece

## Abstract

Loud hydroacoustic sources, such as naval mid-frequency sonars or airguns for marine geophysical prospecting, have been increasingly criticized for their possible negative effects on marine mammals and were implicated in several whale stranding events. Competent authorities now regularly request the implementation of mitigation measures, including the shut-down of acoustic sources when marine mammals are sighted within a predefined exclusion zone. Commonly, ship-based marine mammal observers (MMOs) are employed to visually monitor this zone. This approach is personnel-intensive and not applicable during night time, even though most hydroacoustic activities run day and night. This study describes and evaluates an automatic, ship-based, thermographic whale detection system that continuously scans the ship’s environs for whale blows. Its performance is independent of daylight and exhibits an almost uniform, omnidirectional detection probability within a radius of 5 km. It outperforms alerted observers in terms of number of detected blows and ship-whale encounters. Our results demonstrate that thermal imaging can be used for reliable and continuous marine mammal protection.

## Introduction

Growing concerns that aquatic noise produced during naval exercises and offshore seismic surveys by the oil and gas industry may be harmful to marine mammals [1], [2], have led an increasing number of regulating agencies to request mitigation measures when issuing permits for such surveys in their nations’ EEZ [3]. The most common measure is to implement a “marine mammal watch”, a team of observers that scans the ship’s environs for signs of presence of marine mammals to trigger a shutdown of the hydroacoustic source when marine mammals are entering a predefined *exclusion zone*.

Marine mammal observers usually scan the ship’s environs for whales using binoculars or the naked eye. Sightings mostly rely on spotting a whale’s blow, which might rise to a height of several meters but is visible for a few seconds only. Hence, in combination with the whales’ prolonged dives, sighting opportunities are rare, which, in addition to the limited field of view and finite attention span of human observers, renders this method personnel-intensive and difficult, even during fair weather and daytime. During darkness it is not feasible.

Use of infrared (IR), i.e. thermal imaging, has been suggested for night-time detection of whales [4]. In thermal imagery, a whale’s blow stands out as a transient, warm feature, at least in front of cold surface waters [5]. However, up to now ship-based IR technology has been unsuitable for detecting whales beyond distances of 150 m. Longer ranges required stable, land-based platforms [6] with tele-optics for enhanced resolution while the field of view was limited to angular segments of 45° or less. Most importantly, detections relied on (retrospective) human screening of the images, which is similarly tedious and error-prone as direct visual observation. Moreover, for mitigation purposes, observations need to cover much of the horizon and to be conducted continuously for weeks to months. Such a mode of operation requires automatic detection capabilities, which are introduced and validated in this paper. However, the system described herein is not intended to operate in an unsupervised mode, but to reliably alert a marine mammal observer about the likely occurrence of any whale blow in the ship’s environs, while facilitating its immediate verification and documentation.

## Materials and Methods

The infrared detection system consists of a thermal imaging device (FIRST-Navy) mounted on an actively stabilized gimbal (both by Rheinmetall Defence Electronics, Germany) in combination with a custom data acquisition and processing software (Tashtego, http://tashtego.org). The cryogenic sensor is cooled to 84 K using a Sterling cooler. It scans 360° horizontal×18° vertical at 5 revolutions per second, providing a 5-Hz video stream of the thermal field of the ship’s environs at horizontal and vertical resolutions of 0.05°/pixel and 0.03°/pixel, respectively. The sensor is installed 28.5 m above the sea surface on-board *RV Polarstern* and was deployed for a total of 280 days during 7 expeditions to the Arctic and the Southern Ocean. All expeditions to the Southern Ocean were conducted under Permits from the German Environmental Agency with following identification codes: I 3.5 - 94003-3/218; I 3.5 - 94003-3/238; I 3.5 - 94003-3/247; I 3.5 - 94003-3/278; I 3.5 - 94003-3/273. Expeditions to the Arctic and Atlantic did not require permitting from ethical committees, since the technology used is strictly passive (i.e. observational).

**Figure 1:**
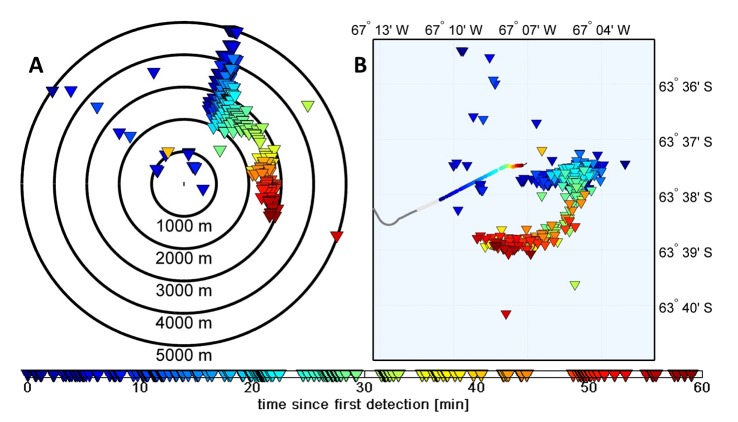
Mapping of blows by a pod of humpback whales. A: Whale blow locations (triangles) relative to the (moving) ship (ship is at center of concentric circles). B: Map of corresponding geo-referenced ship positions (dots) and blow locations (triangles). Color indicates time after first detection.D.

Ship-whale-distances are calculated by spherical triangulation [7] using the angle below the horizon (resolved to ±0.05°), providing unbiased ranges better than 12% accuracy (at 5 km) of the ship-blow distance, i.e. better than achievable by the use of handheld binoculars (Figure S1). Detailed geo-referenced maps of ship-whale encounters are derived in conjunction with bearing information (available to within 0.1°) and the ship’s navigational data, allowing for inferences on the whales’ behavioral response, respiration rates, and dive cycles as exemplified in Figure 1.

**Figure 2:**
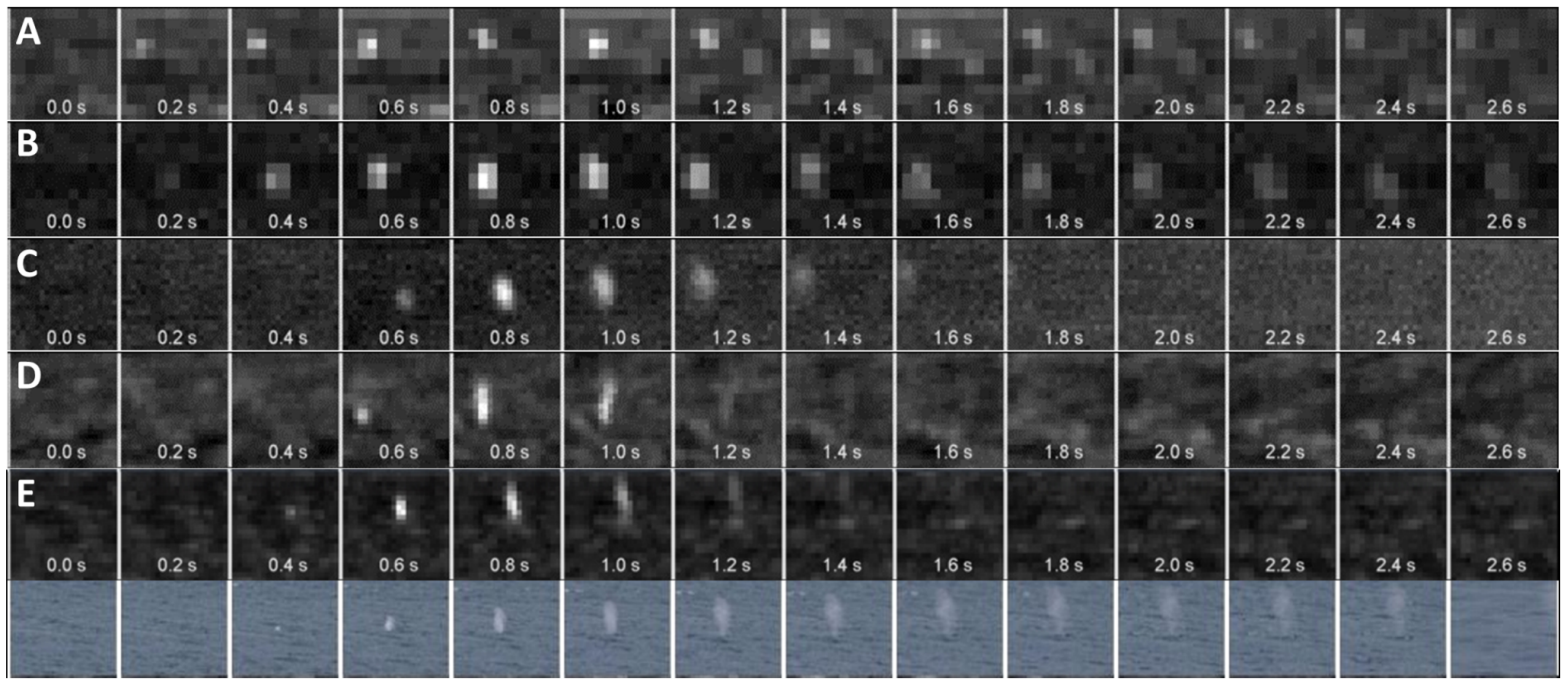
Night-time thermographic video snippets (at 0.2 s resolution) of whale blows: A) 24.03.2012 00:07; 61.11°S 56.36°W; T_water_ = 1.3°C; T_air_ = −1.7°C; r = 3608 m; B) 28.03.2012 03:27; 61.88°S 60.29°W; T_water_ = 1.4°C; T_air_ = 2.3°C; r = 3608 m; C) 29.12.2011 01:06; 56.49°S 00.00°E; T_water_ = −0.8°C; T_air_ = −0.5°C; r = 1116 m; D) 01.01.2012 02:38; 43.96°S 07.44°E; T_water_ = 8.8°C; T_air_ = 8.7°C; r = 879 m; Figure 2E: Day-time thermographic (top) and visual (bottom) video snippets of an automatically detected whale blow: 28.12.2011 14:41; 58.65°S 0.02°E; T_water_ = −1.5°C; T_air_ = −0.6°C; r = 1072 m;

To develop an automatic detection system for whale blows from thermal images, we started with retrospective human screening of thermographic video recordings from multiple expeditions to extract a set of sample blows. On this basis, an automatic detection algorithm was designed to detect temporal contrast changes identified as whale blows (Figure 2) in a standard *detector/classifier* approach, using multi-scale sliding windows [8]–[11]. The *detector* identifies significant thermal anomalies using a modified short-term-average/long-term-average algorithm (STA/LTA) [12] (Figure S2), the *detector* identifies significant thermal anomalies which are then classified as a *blow* or a *no-blow* event. Computer classification of pertinent video snippets is performed after reduction of dimensionality through spatial and temporal centering and clipping through an Eigenimage algorithm [13], [14] before applying a predetermined SVM-based (Support Vector Machine) *classification model* [15]. Training of the SVM is conducted under supervised learning from 120 manually validated *blow* and 1400 *no-blow* events selected from a period of 21 days from expedition ANT-27.2 [16], covering different environmental conditions, distances, and whale species.

Comparisons at the encounter level were based on visual sighting data collected by MMOs conducting continuous transect counts [17] during a 31-day long expedition, ANT-28.2 [18], from Cape Town to Antarctica and back. Observers recorded sighting time and, if possible, species, but not distance. Observations were conducted from the ship’s bridge for a total on-effort time of 299 hours, with 34 ship-whale encounters logged. For 3 of these encounters, the IR system was not operational, and for another 5 encounters, sighting records lacked time information of adequate precision (i.e. to the minute), resulting in a total of 26 visual sightings suitable for comparison with the automatic detection system. Concurrent (within +/−10 minutes) visual and IR encounters were considered *detected encounters*, all other *missed encounters*.

To study the impact of distance on the detection algorithm’s performance, dedicated cue-based comparisons were conducted on the basis of two periods of IR recordings of 50 and 60 min duration collected on 13 and 16 January 2011 during expedition ANT-27.2. Concurrent visual observations provided 303 to-the-second recordings of whale blows, which allowed us to match blows from visual observations with blows from IR recordings. From the IR images, we also determined direction and distance of each automatically detected blow.

An automatic thermal detection was considered a *true positive* if it occurred 3 seconds prior or after a “concurrent” visual cue, or if it was unambiguously validated by retrospective human screening of the IR footage. The latter criterion is indispensable to properly classify blows that were missed by the observer. *False negatives* (events missed by the detection algorithm) were attributed to visual sighting records that lacked matching automatic detections within ±3****s of the sighting. Blows overlooked by both human observers as well as the automatic detector are (unavoidably) left unconsidered. When multiple blows occurred within 1****s, the observers could only record one. Hence, in favor of doubt, all automatic thermal detections within that single second were counted as observed by the MMO.

Visual (including IR) detection of whales depends on them being at the sea surface, a factor known as availability bias. A simple numerical model was developed to estimate this bias for a set of detection radii, average dive/surface times and ship speeds. The model assumes randomly distributed, horizontally stationary animats (animal agents, Figure S3) which are presumed to surface and dive according to a binary dive function extracted from surface and subsurface periods as published in the literature (Table S1). The initial vertical position of each animat is based on its dive state (*at surface*/*subsurface*) at a randomly chosen point in time t_0_ during its dive cycle. Model time progresses in 1-minute steps, Δt, with each animat’s vertical position (*at surface*/*subsurface*) being updated according to its dive function at t_0_+Δt.

Concurrently, the ship transects the model space diagonally at a speed of 4.5 knots, a value typical for seismic surveys. The animat is considered detectable from the moving ship if it is at the surface and inside an assumed detection radius r_detection_. It is considered undetectable if it is diving or outside r_detection_. To estimate, in the context of marine mammal mitigation, the likelihood of detecting an animal before it is within the exclusion zone, which is moving with the seismic source (here r_exclusion_ = 500 m, centered 500 m behind the ship), the model algorithm applies the following classification: An animat is considered

*detected timeously*, if it surfaced within the detection zone before being within the exclusion zone;

*missed*, if it is within the (moving) exclusion zone before having surfaced inside the detection zone.

The probability for detecting an animat *timeously* is then calculated by dividing the number of animats *detected timeously* over the total number of animats blanketed by the (moving) exclusion zone.

## Results

The automatic thermographic whale detection system introduced in this study continuously scans for whale blows in the environs of a ship operating offshore. By human screening, several hundred whale blows were unambiguously identified within a range of 8 km, with most of the blows originating from a distance of less than 4 km range (Figure 2). Using the automatic detection system on data from 7 expeditions, we identified more than 4500 whale blows at distances of up to 5500 m. These blows occurred over the course of more than 300 ship-whale encounters, during both night and day (defined as period between civil twilight), and for a wide range of environmental conditions, with sea surface temperatures ranging between −1.8 and +22.7°C and wind speeds between 0 and 7 Bft.

**Figure 3:**
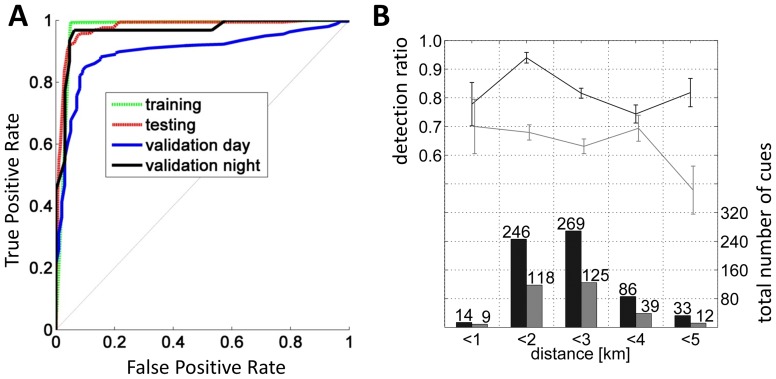
Detection performance and efficiency. A: Cue-based classifier ROC curves for training, testing and two (day and night) validation datasets. AUC values: Training: 0.99; Testing: 0.98; Validation day: 0.90; Validation night: 0.98**.** B: Lines with error bars: Proportion of successful automatic detections of all visually detected blows (black), and proportion of visually detected blows of all automatic detections (grey) versus distance (bin width of 1 km). Errorbars give the standard error. Bar plot: Number of automatic (black) and visual (grey) detections versus distance.

A key component of the automatic detection system is the *classifier*, which selects probable whale blows from a multitude of thermal anomalies provided by the *detector*. The *classifier’s* efficiency is described quantitatively by Receiver-Operator-Characteristic (ROC) curves for false positive and false negative detection events (Figure 3A, green and red curves curves). The resulting Area Under the Curve (AUC) value, which is an integral measure of the reliability of the *classifier*, was 0.99 for the training data set, and 0.98 for the test data set. These AUC values however likely overestimate *classifier* performance; although the test data set and the training data set do not overlap, both are drawn from the same ship-whale encounters (collected during expedition ANT-27.2) and therefore represent similar environmental conditions and encounter ranges. To avoid this bias, we compiled an independent validation data set of 1074 manually classified thermal recordings, including data from a different expedition (ANT-28.4) [19], and evaluated recordings separately for day and night. *Classifier* performance was better at night (AUC = 0.98) than during day (AUC = 0.90), probably due to the lack of glare in the night-time images (Figure 3A, black and blue curves).

To evaluate the overall performance of the automatic detection system, comparisons with the “industry standard” of dedicated, trained visual observers were performed. Analyses were conducted using independent data sets at two different levels: a) at the encounter level (using ANT-28.2 data) and b) at the cue (i.e. blow) level, using ANT-27.2 data.

Of 26 visually recorded encounters during ANT-28.2, the IR system automatically detected 24. One of the two *missed encounters* occurred during high wind speed (11.5 ms^−1^) and presence of an unusually high number of growlers (floating blocks of ice), which generated intense changes in contrast throughout the image. This probably led to a high STA/LTA threshold, leaving the blow undetected. Alternatively, the blow might have been distant, as indicated by the visual observers being unable to identify the animal’s species. The second *missed encounter* was that of a blue whale which surfaced right in front of the ship (pers. comm. D. Verbelen) and probably was too close (<110 m) to be within the field of view of the IR camera.

During the same expedition, the total number of infrared based encounters amounts to 85. Of these, 45 IR-based encounters occurred when the MMOs were on-effort, logging a total of 24 concurrent sightings (53%). The remaining 40 IR-based encounters occurred when the visual observers were off-effort. For 45% of (virtual) 2-hour watches, no false positives occurred; for more than 90% of the 2-hour watches less than 30 *false positives* occurred.

To determine range dependent detector efficiency, cue-based comparisons were performed. Each analysis period commenced with the first visual spotting of a blow, ensuring that the MMOs were alerted. Within the 0–5 km range, the algorithm detected 82% of all blows (303) sighted by the alerted observers, exhibiting a rather range-unspecific detection efficiency between 75 and 95% (Figure 3B, black data). The remaining blows were discernible in the thermographic recordings, but were too faint or unspecific to be picked up by the automatic detection algorithm. Conversely, the observers spotted about 63% of all events that were detected automatically within a 5 km range (Figure 3B, grey data). The average *false positive* (false alert) rate of the IR system was about 6 per hour, with false alerts frequently being caused by nearby birds.

## Discussion

This study introduces a ship-based implementation of thermal imaging for automated marine mammal detection, consisting of a spinning IR camera and an algorithm that detects whale blows on the basis of their thermal signature. The system detected 92% of all visually logged ship-whale encounters during expedition ANT-28.2 and 82% of cues recorded by a team of visual observers during ANT-27.2 (Figure 3). During these expeditions, the system detected about twice (2.5- and 2.1-fold) as many encounters and cues, respectively, as recorded by the MMOs, with false positive rates of less than 1 per 4 minutes occurring for the majority (>90%) of virtual 2-hour watches. On occasion, false positive rates exceeded 1 per minute, due to flocks of birds or presence of growlers during high sea states. However, the system *per se* is conceived as a “bell-ringer” rather than to operate completely unsupervised, alerting the MMO to likely whale blows in the ship’s environs while providing instant playback and documentation of the thermographic recordings. This allows the operator to easily verify the event and quickly determine whether a shut-down request should be issued or not.

Night and day detections rates are comparable. On expedition ANT-28.2 during 76 hours of nighttime observations, 7 encounters were detected by the IR system, resulting in a detection rate of 0.09 encounters/hour while the daytime encounter rate was 0.13 encounters/hour, based on 583 daytime hours. Generally, nighttime performance exceeds daytime performance due to the lack of glare and diffuse reflections, as indicated in the increased nighttime AUC values of Figure 3A (black vs. blue curve).

As with any optical detection system, its performance varies with environmental conditions such as fog, precipitation, sea state, glare, water- and air-temperatures and ambient brightness (insolation). Due to the currently available limited number of visual (i.e. reference) sightings, a statistically significant analysis of system performance in relation to these parameters cannot yet be performed. However, some general trends are already discernible. During ANT-28.2, the number of detections did not degrade up to wind speeds of 7 Bft (corresponding to sea state 6); Wind speeds higher than 7 Bft occurred for only a brief period (<12 h) during which no ship-whale encounter was detected. Detections also occurred at water temperatures of up to 23°C, yet sampling effort was heavily biased towards polar water temperatures with only 5 encounters having occurred in waters warmer than 15°C. Air temperatures are irrelevant to system performance as (dry) air is quasi-transparent in the LWIR (8–12 µm) band used. Contrastingly, fog may significantly compromise system performance. Depending on droplet size, visibility in the LWIR band has been noted to be equal or better than in the visual (0.3–0.7 µm) band. Fog, rain and snow occurred rarely (visibility was less than 500 and 1000 m for only 0.5 and 2% of the expedition, respectively), representative of typical Southern Ocean conditions during austral summer. Glare resembles a clutter of warm anomalies in thermographic images, resulting in high local contrasts. This raises the STA/LTA threshold, rendering the detection of blows less likely. However, the field of glare in the IR image is significantly narrower than for visual observers, as the detector only considers the local contrast of each analyzed tile (between 1 and 3° horizontal field of view, FOV), rather than that of the human (i.e. binocular’s) field of view (8°FOV).

As yet, the upper limit of sea surface temperatures and the lower limit of mammal sizes allowing reliable blow detection remain unknown. Our results were obtained for a limited range of environmental conditions and species, with sea surface temperatures predominantly between −1.8 to +10°C, wind speeds below 7 Bft, and species consisting mainly of humpback (*Megaptera novaeangliae),* minke (*Balaenoptera bonaerensis*) and fin whales (*Balaenoptera physalus*). A comprehensive evaluation of the algorithm’s efficiency for other whale species and pinnipeds, and for higher sea surface temperature and wind speeds, including their cross-dependencies, requires further studies which are in planning. Meanwhile, our results demonstrate that the IR systems works well for large whales in the subpolar and polar oceans, and provides a major breakthrough for night time detection.

**Figure 4:**
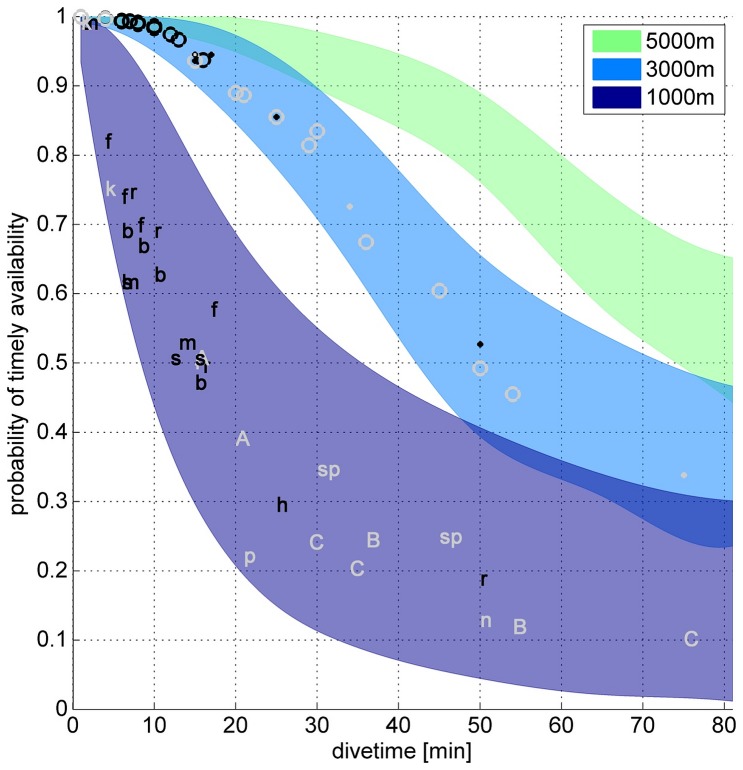
Timeous availability. Probability of a whale being at the surface within a detection radius r before it is within the exclusion zone (radius 500 m, acoustic source towed 500 m behind the ship, see Figure S3) as a function of diving time. Ship speed is assumed to be 4.5 knots. Colored areas indicate different maximum radii over which whales can be reliably detected (1, 3 and 5 km). Upper limits of filled areas correspond to the maximum, lower limits to the minimum known surface times. Mean values for various whale species are indicated by letters: A = Arnoux’s beaked whale, B = Blainville’s beaked whale, b = blue whale, C = Cuvier’s beaked whale, f = fin whale, h = humpback whale, k = killer whale, m = minke whale, n = northern bottlenose whale, p = pilot whale, r = right whale, s = sei whale, sp = sperm whale. Letters are only displayed for a detection radius of 1 km. Within in the blue (3 km) area, circles and dots vertically aligned with letters indicate whether the underlying data represents single measurements (dots) or group averages (circles). Light grey symbols represent odontocetes, black symbols mysticetes.

The reliability of visual or thermographic observations for cetacean mitigation is strongly dependent on the ratio of a whale’s surface versus dive times, a factor known as availability bias. Modeled availability biases range from 99% for whales exhibiting dive times of 10 min (baleen whales), over 65% for dive times of 40 min (sperm whale), to 45% for dive times of 60 min (beaked whales). The degree to which availability bias impedes a *timeous detection*, that is, a whale’s detection before it enters the exclusion zone, depends further on the radius and relative position of the detection and exclusion zone and the ship’s speed. Longer dive times in combination with a detection radius below 3 km lead to a dramatic decrease in the probability that a whale can be detected *timeously* (Figure 4, Figure S3). Baleen whales, for example, are very likely (>90%) to have surfaced within the IR system’s detection range before entering the exclusion zone, whereas whales with long dive times (odontocetes in particularly) stand a reduced chance for being detected *timeously* (40–70%). The modeled values likely represent conservative (low) estimates, as the possibility of avoidance responses of the animals to loud sounds [20]–[23] was disregarded in the model. In addition, whale pods with asynchronous diving patterns present multiple detection opportunities, increasing the likelihood and of being detected before entering the exclusion zone.

Further protection might be achieved by concurrent passive acoustic monitoring of animal sounds [24]. However, quantitative and comprehensive studies of the detection probability of passive acoustic monitoring are largely lacking. With the exception of odontocetes, which emit clicks during foraging dives, the vocalization behavior of most species at the gender and contextual level is insufficiently understood for quantitative estimates of acoustic detectability. By contrast, whales need to respire regularly, rendering visual or thermographic detection methods reliable once the detection bias is minimized, as achieved by the system described herein. Further progress in sensor technology, such as the availability of multiple band (far and mid wavelength IR and visual) sensors and higher image resolution can be expected to further increase detection reliability and therefore whale protection.

The IR system presented here has additional benefits. It provides precise and reproducible distance and bearing information which can be used to study the response of whales to acoustic exposure with regard to locomotive behavior, respiration rates, and dive cycles (Figure 1). Automated blow detection can be coupled with acquisition of additional visual imagery for species identification and morphometric analyses, an approach currently under development. The increased use of such systems will eventually result in a large number of well documented encounters, providing urgently needed, statistically robust data resolved at the species and contextual levels [23]. With regard to marine mammal mitigation applications, the real-time detection and tracking capability of thermal imaging methods allows for fast and correct decisions, day and night, throughout seismic surveys or naval activities. In particular, the IR system’s ability to concurrently detect multiple whales allows for full situation awareness, even in the presence of many whales.

## Supporting Information

Figure S1 Absolute (A) and relative (B) error estimation of image and binocular based distance calculation. It is assumed that the vertical position of a whale in the thermal image is determined with an accuracy of ±1 pixel and with ¼ reticule accuracy using binoculars. Due to the spherical triangulation used to calculate the distance, this results in a distance dependent error. Red color indicates upper, blue color indicates lower error boundaries. For distances less than 5 km, relative errors are within 12%. (TIF)

Figure S2 Schematic of the STA/LTA algorithm. The example shows the V-shaped blow of a humpback whale. The black curve shows the short term contrast average (STA), the red curve the long term contrast average (LTA) computed from the sequence of snippets above. Blue and pink windows indicate the number of images used to calculate STA and LTA respectively. The blue curve indicates the adaptive threshold (AT) as computed from the right hand side of equation (1). (TIF)

Figure S3 Schematic of ship position, exclusion zone and detection zone. For the animat simulation model (see Figure 4), the sound source was assumed to trail 500 m behind the ship. (TIF)

Table S1 Surface and dive times used for animat model, as taken from the literature. (DOC)

Text S1 Detailed description of detection and classification algorithms. (DOC)
