# Supplementary figures and images for: Automatic Round-the-Clock Detection of Whales for Mitigation from Underwater Noise Impacts
Source: PLoS One. 2013 Aug 12;8(8):e71217. doi: 10.1371/journal.pone.0071217 (PMC3741354; doi:10.1371/journal.pone.0071217)

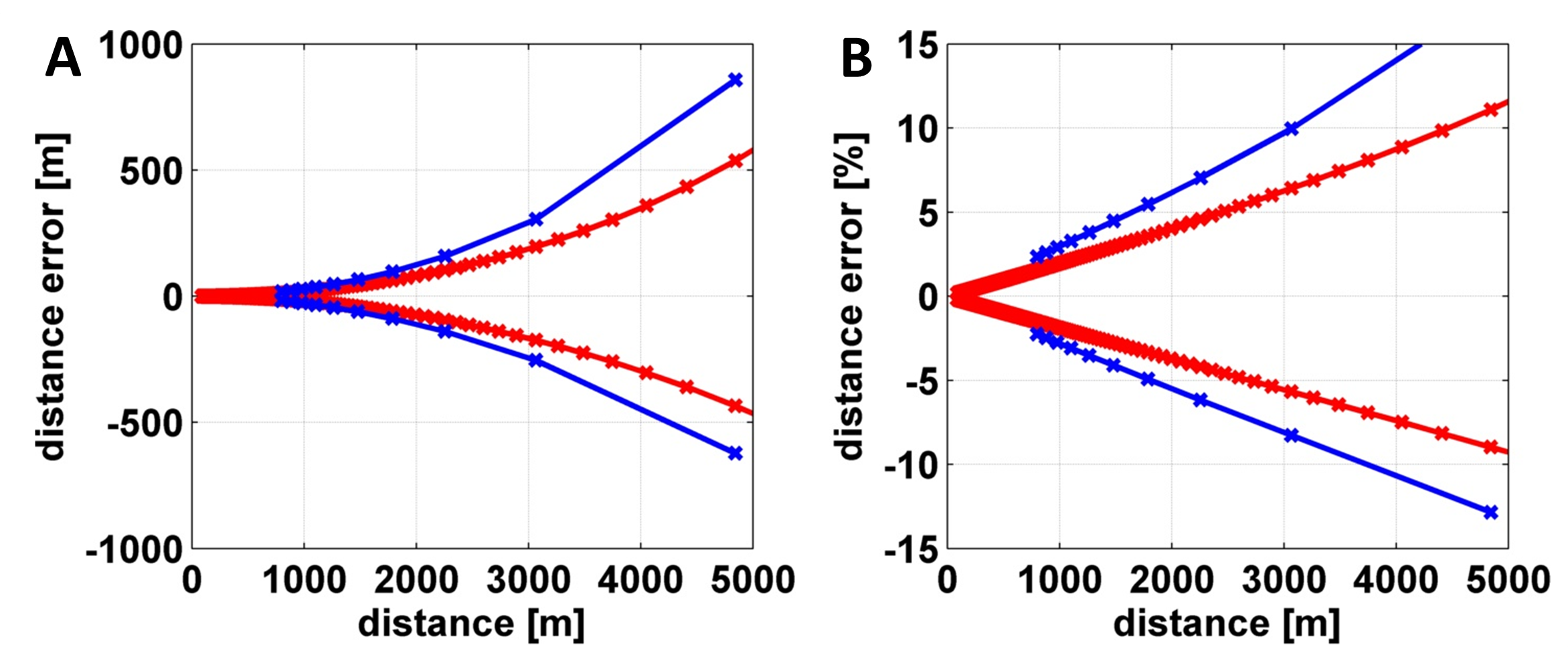

Supplement: Figure S1 — (TIF) [file pone.0071217.s001.tif]

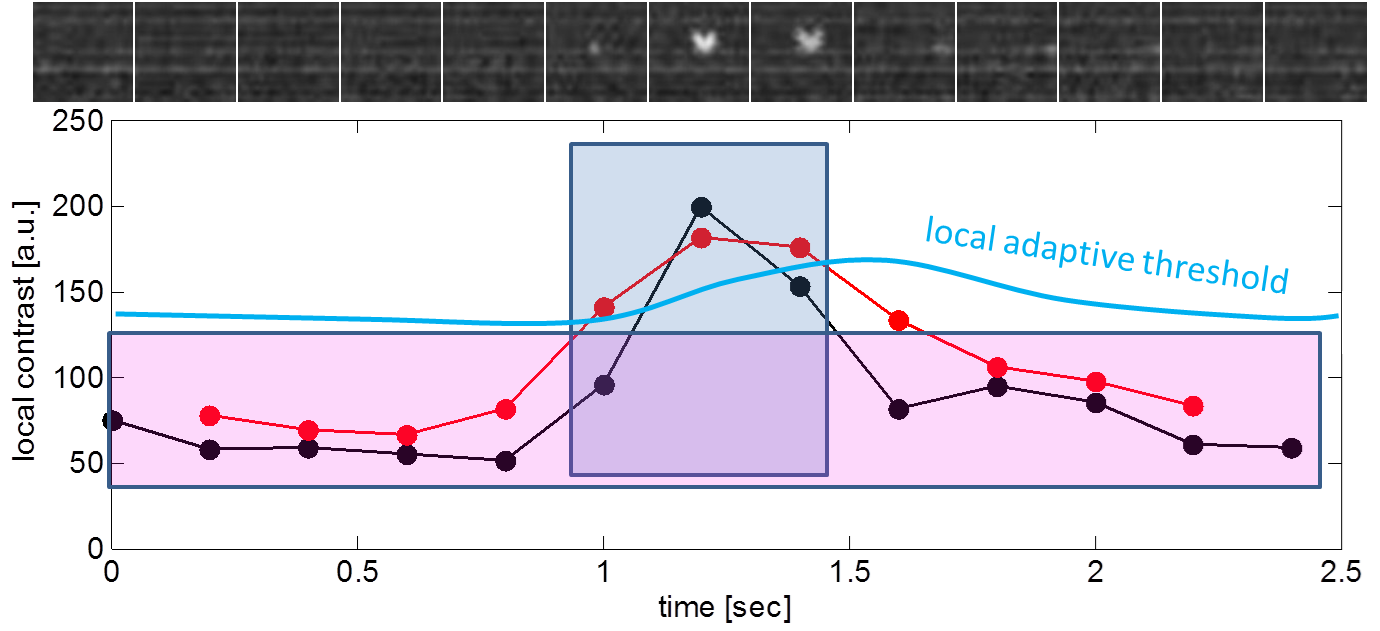

Supplement: Figure S2 — (TIF) [file pone.0071217.s002.tif]

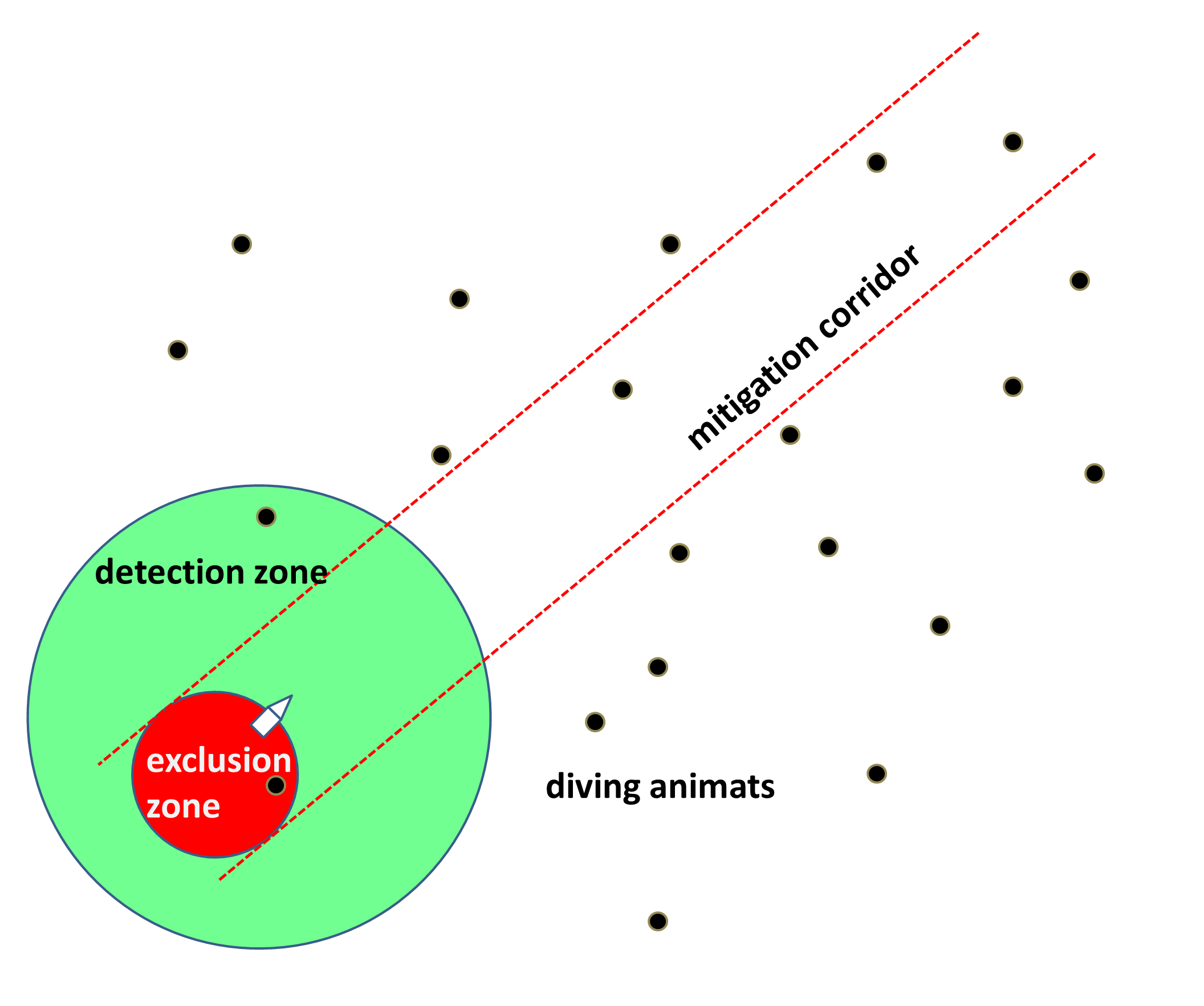

Supplement: Figure S3 — (TIF) [file pone.0071217.s003.tif]
