# Supplementary material for: Automatic Round-the-Clock Detection of Whales for Mitigation from Underwater Noise Impacts
Source: PLoS One. 2013 Aug 12;8(8):e71217. doi: 10.1371/journal.pone.0071217 (PMC3741354; doi:10.1371/journal.pone.0071217)
Supplement: Table S1 — (DOC) [file pone.0071217.s004.doc]

| Table S*1*: Diving times and surface times of cetacean species.  “single” = analysis of single dives;  “max” = maximum values of multiple dives from several individuals;  “Ø” = average;  “median” = median values of multiple dives from several individuals;  “range” = minimum and maximum values of several dives from several individuals; | | | |
| --- | --- | --- | --- |
| **Species** | **diving time  [min]** | **surface time [min]** | **reference** |
| Blue whale Ø max max | 8 ± 2 14.7 - | - 2-3 - | [25] [25] [26] [27] |
| Fin whale single single max Ø | 30 10 16.9 6 ± 2 | *-* ≈10[[1]](#footnote-2) *-* ≈3^*^ | [28] [29] [25] [25] [30] |
| Sei whale single  single range | 20-30sek 15 15 0,5-12 | - - - < 1 | [31] [31] [28] [32] |
| Minke whale[[2]](#footnote-3) | 0.6- 13 |  | [33] [34] |
| Humpback whale Ø Ø | 21 25.7 | - - | [28] [35] |
| S. right whale |  |  |  |
| Sperm whale Ø Ø range | 40 45 30-45 | 10 10,5 (day); 7 (night) 8 | [36] [37] [38] |
| Arnoux’s bkd. w. Ø single | 15-20 70 |  | [39] [40] |
| Southern bottlenose whale range Ø |  | 3.7 s | [41] |
| Killer whale  Ø max | 1-4 12 | 5-10 sec between dives (Usually long times at surface while not diving) | [42] [43] |
| Long-finned pilot whale  Ø max max | 15-21 26  18 | long times in the upper 16 m of the water column during day | [42] [44] [45] |
| N. bottlenose whale range | 25,25- 70,50 | - | [46] |
| Cuvier’s-BW max range | 34 – 75[[3]](#footnote-4) | some sec. | [47] [48] |
| Blainville’s-BW max range  median | 36 - 54^‡^  29 | some sec.  2 | [47] [48]  [49] |
| N. right whale max range single | 50 7,83 – 16,32 ≈12^*^ | - 4,54 – 11,08 ≈3^*^ | [28] [50] [51] |

25. Croll DA, Acevedo-Gutierreza A, Tershy BR, Urban-Ramırez J (2001) The diving behavior of blue and fin whales: is dive duration shorter than expected based on oxygen stores? Comparative Biochemistry and Physiology Part A 797-809.

26. Calambokidis J, Chandler T, Schlender L, Steiger GH, Douglas A (2003) Research on humpback and blue whales off California, Oregon and Washington in 2002. Olympia, WA 98501: Cascadia Research.

27. Oleson E, Calambokidis J, Burgess WC, McDonald MA, Leduc CA, et al. (2007) Behavioral context of call production by eastern North Pacific blue whales. Marine Ecology Progress Series 330: 269-284.

28. Schreer JF, Kovacs KM (1997) Allometry of diving capacity in air-breathing vertebrates. Canadian Journal of Zoology 75: 339-358.

29. Panigada S, Zanardelli M, Simonpetietro C, Jahoda M (1999) How deep can baleen whales dive? Marine Ecology Progress Series 187: 209-311.

30. Goldbogen JA, Calambokidis J, Shadwick RE, Oleson EM, McDonald MA, et al. (2006) Kinematics of foraging dives and lunge-feeding in fin whales. J Exp Biol 209: 1231-1244.

31. Gambell R (1985) Sei whale *Balaenoptera borealis* (Lesson,1828). In: Ridgway SH, Harrison R, editors. Handbook of Marine Mammals, Vol 3: The sirenians and baleen whales. London: Academic Press. pp. 155-170.

32. Leatherwood S, Goodrich K, Kinter AL, Truppo RM (1982) Respiration patterns and 'sightability' of whales. Rep Int Whal Commn 32: 601-613.

33. Stockin K, Fairbairns R, Parsons E, Sims D (2001) Effects of diel and seasonal cycles on the dive duration of the minke whale (*Balaenoptera acutorostrata*). Journal of the Marine Biological Association of the United Kingdom 81: 189-190.

34. Øien N, Folkow L, Lydersen C (1990) Dive time experiments on minke whales in Norwegian waters during the 1988 Season. Rep Int Whal Commn 40: 337-341.

35. Baird RW, Ligon AD, Hooker SK (2000) Sub-surface and night-time behavior of humpback whales off Maui, Hawaii: A preliminary report. # 40ABNC050729 # 40ABNC050729. 19 p.

36. Papastavrou V, Smith SC, Whitehead H (1989) Diving behaviour of the sperm whale, *Physter macrocephalus*, off the Galapagos Islands. Canadian Journal of Zoology 67: 839-846.

37. Watkins WA, Daher MA, DiMarzio NA, Samuels A, Wartzok D, et al. (2002) Sperm whale dives tracked by radio tag telemetry. Marine Mammal Science 18: 55-68.

38. Amano M, Yoshioka M (2003) Sperm whale diving behavior monitored using a suction-cup-attached TDR tag. Marine Ecology Progress Series 258: 291-295.

39. Balcomb KC (1989) Baird's beaked whale *Berardius bairdii* Stejneger, 1883: Arnoux's beaked whale *Berardius arnuxii* Duvernoy, 1851. In: Ridgway SH, Harrison R, editors. Handbook of Marine Mammals. London: Academic Press. pp. 261-288.

40. Hobson RP, Martin AR (1996) Behaviour and dive times of Armoux's beaked whales, *Berardius arnuxii*, at narrow leads in fast ice. Canadian Journal Zoolgy 74: 388-393.

41. Kasamatsu F, Joyce GG (1995) Current status of odontocetes in the Antarctic. Antarctic Science 7: 365-379.

42. Baird RW, Hanson MB, Ashe EE, Heithaus MR, Marshall GJ (2003) Studies of foraging "southern resident" killer whales during July 2002: dive depth, bursts in speed, and the use of a "crittercam" system for examing sub-surface behavior. Seattle, WA: Report submitted to the National Marine Mammal Laboratory.

43. Baird RW, Dill LM, Hanson MB. Diving behaviour of killer whales; 1998 January 1998; Monaco. pp. 9.

44. Nawojchik R, St Aubin DJ, Johnson A (2003) Movements and dive behavior of two stranded, rehabilitated long-finned pilot whales (*Globicephala melas*) in the Northwest Atlantic. Marine Mammal Science 19: 232-239.

45. Heide-Jørgensen MP, Bloch D, Stefansson E, Mikkelsen B, Ofstad LH, et al. (2002) Diving behaviour of long-finned pilot whales *Globicephala melas* around the Faroe Islands. Wildlife Biology 8.

46. Hooker SK, Baird RW (1999) Deep-diving behaviour of the northern bottlenose whale, *Hyperoodon ampullatus* (Cetacea: Ziphiidae). Proceedings of the Royal Society 266: 671-676.

47. Johnson M, Madsen PT, Zimmer WMX, Aguilar Soto N, Tyack PL (2004) Beaked whales echolocate on prey. Biology Letters 271: S383-S386.

48. Tyack PL, Johnson M, Aguilar Soto N, Sturlese A, Madsen PT (2006 ) Extreme diving of beaked whales. Journal of Experimental Biology 209 4238-4253.

49. Barlow J, Gisiner R (2006) Mitigating, monitoring and assessing the effects of anthropogenic sound on beaked whales. J CETACEAN RES MANAGE 7: 239-246.

50. Baumgartner MF, Mate BR (2003) Summertime foraging ecology of North Atlantic right whales. Marine Ecology Progress Series 264: 123.

51. Nowacek DP, Johnson MP, Tyack PL (2004) North Atlantic right whales (*Eubalaena glacilis*) ignore ships but respond to alerting stimuli. Proceedings of the Royal Society London B 271: 227-231.

1. Taken from graph. [↑](#footnote-ref-2)
2. Values given for *B. acutorostrata* [↑](#footnote-ref-3)
3. Taken from their figures 1 and 2, respectively. [↑](#footnote-ref-4)
