# Supplementary material for: Automatic Round-the-Clock Detection of Whales for Mitigation from Underwater Noise Impacts
Source: PLoS One. 2013 Aug 12;8(8):e71217. doi: 10.1371/journal.pone.0071217 (PMC3741354; doi:10.1371/journal.pone.0071217)
Supplement: Text S1 — (DOC) [file pone.0071217.s005.doc]

Detection algorithm

Thermal images are acquired over a 1GBit Ethernet cable using the UDP protocol. Images are stored in a 5‑frame, shared memory ring-buffer and read by the analysis software. Prior to the image processing procedure, pixel intensities are dynamically flat-field corrected at each sensor revolution by imaging over a copper plate with an even temperature.

The detector and classifier operate on multi-scale, sliding windows (tiles) [8-11] of the full image, which renders the detection system insensitive against changes of global contrast. Tile size depends on its vertical position within the image and varies from 11x11 to 63x63 pixels (11, 23, 43, 63), as the vertical position in the tile corresponds to horizontal distances and therefore different apparent sizes of a blow. Tiles overlap by 50% to avoid splitting signatures located near a tile’s edge. Candidate tiles containing suspected blow events are selected by the *detector* on the basis of each tile’s temporal change in contrast (thermal anomaly). The *detector* first normalizes the intensity to the tile’s mean over time before using a STA/LTA (short-term-average over long-term-average) [12] algorithm as a measure of change of contrast over time (Figure S2). The tile’s STA/LTA value has to meet the following condition to be considered a candidate tile:

$$\frac{\langle\max(I(t))-\min(I(t))\rangle_{dt=-1}^{1}}{\langle\max(I(t))-\min(I(t))\rangle_{dt=-5}^{5}}>n\cdot\langle\max(I(t))-\min(I(t))\rangle_{dt=-30}^{0}+1.5\cdot\sigma(\langle\max(I(t))-\min(I(t))\rangle_{dt=-30}^{0})$$

The right hand side defines an adaptive threshold (AT). The values max(*I(t)*) and min(*I(t)*) denote the maximum and minimum pixel intensity within the tile, <…> denote averages over frames specified by the numbers after the brackets, σ denotes the standard deviation, and the factor n is selected dynamically, keeping the number of resulting candidate tiles constant to facilitate real-time processing capability. This implies that areas of persistent high contrast (e.g. as occurring during glare and high numbers of small ice blocks, i.e. growlers) will not be considered as candidate tiles, while small contrast changes (i.e. at large distances) will still be detected.

Classification algorithm

Each candidate tile is subsequently *classified* to determine if its thermal signature resembles that of a whale’s blow or not. For classification, all snippets are reduced to a size of 11x11 pixels and 1 sec (5 frames) in time (i.e. 11x11x5 matrices) by spatial and temporal centering and clipping. Classification combines an Eigenimage approach [13,14] to reduce the dimensionality of the tile, and application of a Support-Vector-Machine (SVM) model for subsequent classification [15].

For classifier training, events were obtained from data collected during expedition ANT-27.2 on 11-23 December 2010, and on 8, 19, 20, 21, 23-25, and 27 January 2011, by first running the detector without classifier. Subsequently, the resulting events were annotated manually as *blow* or *no-blow* by human screening. A set of 120 manually validated blow signatures (the true event data set) was extracted and used in conjunction with a set of 1400 “no-blow” (the false event data set) signatures to train the SVM.

For classifier training, the entire event data set (comprising above true and false event data sets) is split evenly and randomly into training and testing data sets. In the next step, a set of eigenvectors is derived from the true training data set using a Principal Component Analysis. Training images are then reconstructed using this set of eigenvectors for the soft margin weighted SVM (nu-SVC) with a RBF Kernel. Kernel parameters are selected applying a grid search algorithm with 5-fold cross validation. The overall performance of the model (*true positives/false positives*) is then measured against the testing dataset. During subsequent applications for the classification of independent snippets (for validation purposes or at sea during operation of the detection system), each candidate snippet is projected onto the training data set’s Eigenbasis and classified according to the pre-calculated SVM-model.

During operation of the system, the only parameter set by the operator is the minimal distance to the hyperplane that is still classified as blow, *P*. According to the setting of this parameter, the operator is alerted whenever an event exhibits a larger distance from the hyperplane. A value of *P = -1* corresponds to classifying every candidate tile as *blow*, while a value of *P =* *+1* implies that only tiles with distances from the hyperplane larger than those of the support vectors set are being selected. It was set to +0.5 for the comparisons presented herein.
